# Use of a digital contact tracing system in Singapore to mitigate COVID-19 spread

**DOI:** 10.1186/s12889-023-17150-0

**Published:** 2023-11-16

**Authors:** Bryan W. K. Chow, Yi Ding Lim, Richard C. H. Poh, Amy Ko, Guo Hao Hong, Steffen W. L. Zou, Joshua Cheah, Shaowei Ho, Vernon J. M. Lee, Marc Z. J. Ho

**Affiliations:** 1 Preventive Medicine Residency Program, National University Health System, Singapore, Singapore; 2 Communicable Diseases Division, Ministry of Health, Singapore, Republic of Singapore; 3 Government Technology Agency, Prime Minister’s Office, Singapore, Republic of Singapore; 4 Saw Swee Hock School of Public Health, National University of Singapore, Singapore, Singapore

**Keywords:** COVID-19, Contact Tracing, Digital Health, Communicable Diseases Control, Epidemiology

## Abstract

**Background:**

Contact tracing has been essential to reducing spread of COVID-19. Singapore leveraged technology to assist with contact tracing efforts using a Bluetooth-based app and token platform called ‘TraceTogether’.

**Methods:**

We reviewed the impact of this system during the country’s Delta and Omicron waves (24 August 2021 to 17 February 2022) to identify differences in number of close contacts and time savings between full automation using TraceTogether alone as compared to manual contact tracing supplemented by TraceTogether. Characteristics of digital contact tracing app or token users were reviewed. Thereafter, the number of close contacts identified by manual and digital contact tracing methods, and the number of confirmed COVID-19 cases among contacts were analysed. The difference in time taken for identification of close contacts was also determined.

**Findings:**

Adoption rate for TraceTogether was high, with 93.3% of cases having a registered device. There was a 9.8 h (34.9%) reduction in time savings for close contacts to be informed using TraceTogether alone compared to manual contact tracing supplemented by TraceTogether. The proportion of close contacts automatically identified through TraceTogether alone and turned positive was 3.6%. For those identified through manual contact tracing supplemented by TraceTogether, this proportion was 12.5% and 6.2% for those served quarantine orders and health risk warnings respectively.

**Interpretation:**

The high adoption rate of ‘TraceTogether’ suggest that digital solutions remain a promising option to improve contact tracing in future epidemics. This may have been through its concurrent use with vaccine differentiated public health measures and policies which engender public trust. There is future potential for utilising such technology in managing communicable diseases to achieve good public health outcomes.

## Introduction

Since the first reported case of COVID-19 infection in Singapore on 23 January 2020, contact tracing has been a key approach to the containment and subsequent reduction of further spread as a mitigation strategy. Initially, this was done manually by public health officers who interviewed each positive COVID-19 case to establish their possible contacts. Subsequently, Singapore developed its own digital contact tracing tools called TraceTogether (TT) and SafeEntry (SE) to supplement manual contact tracing. Similar solutions have been deployed in other countries, although with lower levels of uptake [1–4]. Singapore experienced a surge in Delta variant cases that began towards the end of August 2021, the introduction of Omicron variant in December 2021, and subsequent surge in Omicron variant cases in January to March 2022 [5]. This study reviews the role of the locally-developed TT digital system in supplementing national contact tracing efforts, and compared it against manual contact tracing, particularly during parallel efforts to contain initial Omicron cases. We also discuss its integration with TT-only SafeEntry check-ins, and Singapore’s vaccine differentiated public health and social measures that led to its high uptake and success. This would provide additional insights on the utility and effectiveness of digital contact tracing tools in the management of future pandemics in Singapore and other countries.

## Methods

### TraceTogether, SafeEntry, and vaccine-differentiated public health and social measures

TT is a digital system implemented in Singapore on 20 March 2020, it started as a smartphone application that utilised a custom protocol where participating devices exchanged proximity information whenever an app detects another device with the app installed [6, 7]. On 7 June 2020, a physical device, named the “TT token”, was introduced, primarily for individuals who did not have the TT app installed on their smartphone and for those who did not own a smartphone. The sensitivity and specificity of the TT system has been previously described [8]. All Singapore residents were encouraged to download either the app or obtain a token, and to carry either along when they left their place of residence. Consent to use data for contact tracing was obtained from users on signing up. Confirmed COVID-19 cases were sent an SMS code to upload their TT data of close contacts, and/or their TT tokens were collected at healthcare facilities or their homes, to facilitate contact tracing. Upload of data was not mandatory but strongly encouraged, especially during phone interviews by public health officers doing manual contact tracing.

SE is a check-in system for individuals who visited various public places such as workplaces, schools, shopping malls and restaurants. By physically tapping their mobile phone or TT token, or scanning a Quick Response (QR) code, a place and time stamp would be captured. After 25 days, the data was automatically deleted from TT and SE systems if not used for contact tracing.

From 17 May 2021, the Government announced that higher-risk venues would require mandatory SE check-in. In conjunction, vaccine-differentiated public health measures such as only allowing those who were vaccinated to enter certain high traffic and mask-off locations were introduced to protect high-risk and vulnerable populations from being infected. Under such measures, only fully-vaccinated individuals were allowed entry to selected places such as shopping malls or sports facilities. The same TT system was used to display the person’s vaccination status for venue operators, as part of the check-in process.

### Quarantine orders and health risk warnings

Under the law, public health officers in the MOH had access to cases’ TT and SE data to aid their contact tracing efforts. A close contact would be issued with either a quarantine order (QO) or a “health risk warning” (HRWs). QOs were legally enforceable notices to compel isolation for a set period of time at a specified location (usually their place of residence or in a quarantine facility, such as a dedicated hotel). These were for 14 days, based on the maximum incubation period of the diseases, and were only issued to close contacts of confirmed cases through manual contact tracing.

From 11 October 2021, QOs were replaced with health risk warnings. The exception was a short duration of containment for the Omicron variant from 2 to 26 December 2021 where QOs were reinstated. HRWs were notices that legally required the recipient to self-test negative for COVID-19 with an accredited antigen rapid test kit before leaving their place of residence for a set number of days. They were issued through an automated system if there was 60 min of contact with a confirmed case based on TT. On 17 February 2022, HRWs were replaced by a new isolation and test regime to also signal the country’s move towards living with COVID-19. Table 1 summarises major changes in the shift from QOs to HRWs, relative to Singapore’s Delta and Omicron variant waves.

**Table 1:** Timeline of changes from QO to HRW to cessation of HRW

**Date**	**Description**
24 August 2021 (Start of reference period)	New local cases in Singapore cross 100/day for the first time since early-Aug 2021, heralding the start of the Delta variant wave. Contact tracing was manual, aided by information from the TT system. Contacts were issued quarantine orders.
6 September 2021	First fully automated HRW sent to contacts identified using the TT system. These were individuals recorded as having more than 60 min of contact with a confirmed case.
10 October 2021	Cessation of QO issuance and manual contact tracing for individual cases for the Delta wave.
2 December 2021	First imported case of Omicron variant in Singapore. Start of QO issuance through manual contact tracing, aided by information from the TT system for Omicron cases.
9 December 2021	First local case of Omicron variant in Singapore.
21 December 2021 and 25 December 2021	First major Omicron cluster linked to a gym, and second cluster linked to drinking bars. These were accompanied by an increasing number of unlinked cases in the community.
26 December 2021	Singapore shifts its posture for Omicron to reduction of spread, aligning with the approach for the Delta variant. Cessation of QO issuance and manual contact tracing for individual cases for the Omicron wave
17 February 2022 (End of reference period)	Cessation of HRW issuances, and thus, removal of legal requirements for self-isolation and testing and shortening of overall isolation duration.

### Study period - surge in Delta variant and Omicron variant cases in Singapore

**Fig. 1:**
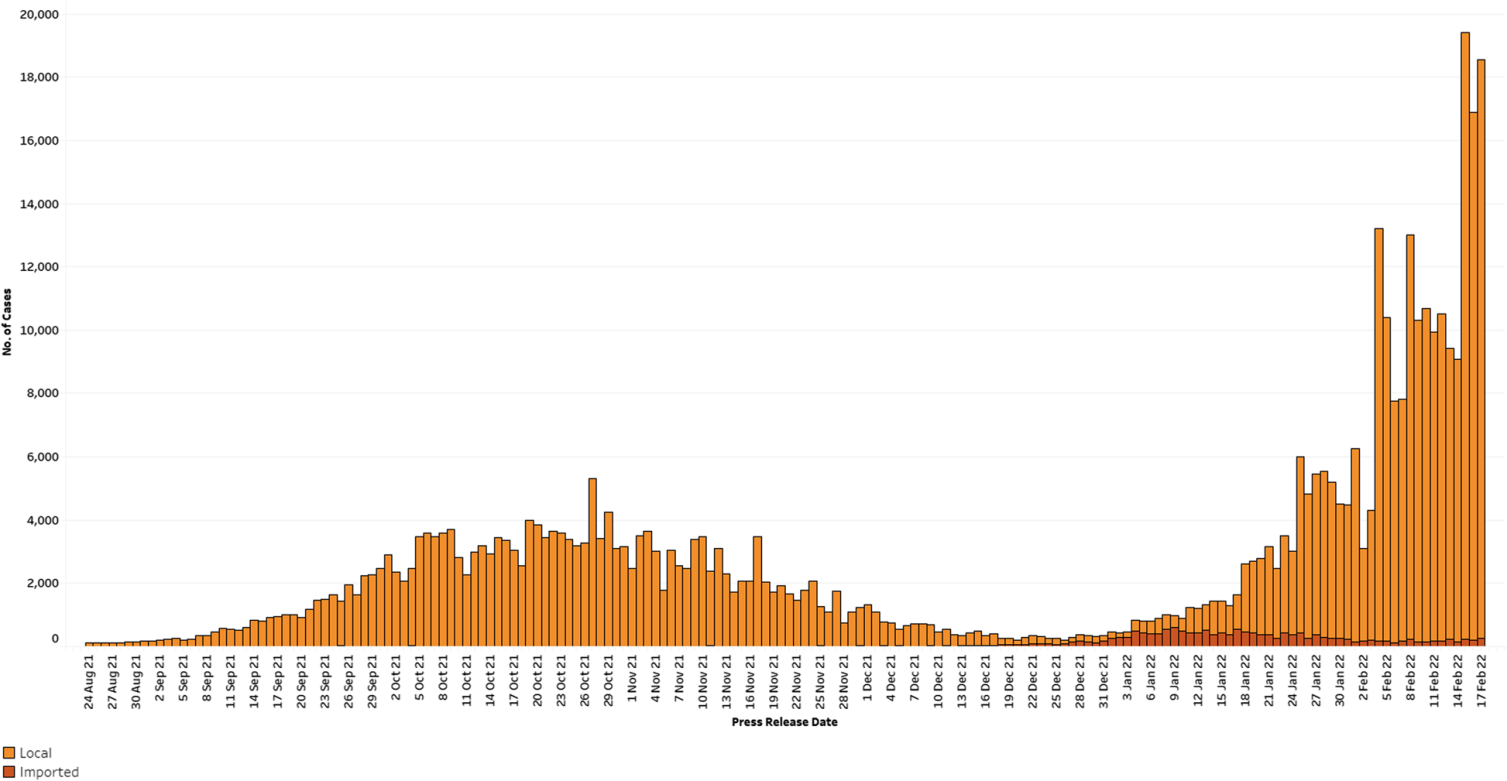
Epidemiology curve for new COVID-19 cases in Singapore

Figure 1 shows the epidemiology curve in Singapore during the study period. 24 August 2021 was used to mark the start of the Delta variant wave and study period. This was when Singapore reported more than 100 local cases per day. New local cases at the peak of this surge was 5,312 on 27 October 2021. This then fell to a low of 109 local cases on 26 Dec 2021 [5].

There was significant community transmission of the Omicron BA.1/2 variant in January 2022, with peak new local cases at 13,046 on 4 February 2022. Given the initial lack of information regarding the Omicron variant, Singapore adopted a containment posture again to prevent transmission as much as possible. With significant community transmission and a better understanding of the Omicron variant, the country shifted to reduction of spread and severe infection thereafter.

The end of the study period was the cessation of HRWs on 17 February 2022, as the country transitioned to a more endemic posture. This reference period from 24 August 2021 to 17 February 2022 coincided with a time period of high TT utilisation within the Singapore resident population, and hence allows for an accurate assessment on its role, especially during periods of high community transmission.

### Definition of COVID-19 cases and contacts

Laboratories and doctors were required to report all suspected and confirmed COVID-19 positive cases under the Infectious Diseases Act. A confirmed case was defined as either having a positive polymerase chain reaction (PCR) result from an accredited laboratory, or a positive antigen rapid test with COVID-19 symptoms as clinically reviewed by a registered healthcare professional.

A central IT platform was used to integrate COVID-19 cases’ names, national identification number, biodata including residential address, age, nationality and gender, travel history, and details on tests conducted, including PCR cycle threshold values. Once uploaded, cases’ TT data and contacts would also be accessible on the central IT platform to facilitate contact tracing and public health decisions, including issuance of QOs or HRWs.

For contact tracing using digital modalities, a close contact was defined as a continuous, uninterrupted 60 min ping between two TT devices within the period of infectiousness prior to positive case confirmation though PCR or documented antigen rapid test.

The lookback period for the TT system was adjusted based on the predominant circulating COVID-19 variant and prevailing context in Singapore. It was 5 days prior to COVID-19 confirmation during Delta wave and 2 days during the Omicron wave. The 5 days was to account for 2 days of infectiousness prior to symptom onset, and 3 additional days to seek medical attention and confirmation through laboratory tests. The adjustment to 2 days thereafter was to account for quicker test turnaround times, especially with the rollout of the point-of-care antigen rapid test.

For manual contact tracing of close contacts supplemented by TT data, the definition of a close contact was an individual who was within 2 m of a positive case, and with at least 30 min of exposure time.

### Inclusion criteria and data sources

All confirmed cases, as well as all Singapore-based residents who signed up for either a TT token or the TT app, registered to their name and unique national identification number, were included in the study. QOs and HRWs issued to close contacts was based on a separate Government-owned IT system managed by the MOH. The analysis was approved and conducted by the MOH under the Infectious Disease Act, with records deidentified prior to the analysis.

### Analysis of data

We examined the utilisation rate of TT and data upload compliance of cases, the number of QOs and HRWs issued to close contacts during the study period, and their origin (manual or digital contact tracing approaches). We also explored the time taken to issue notice of recent contact between manual and digital contact tracing approaches. Finally, we compared the conversion rate of close contacts to cases between digital and manual contact tracing methods.

## Results

### Utilisation rate of TraceTogether system

As of 17 February 2022, there were 5,952,185 unique users for the TT platform. This comprised 1,481,628 TT app-only users, 724,594 TT token-only users, and 3,745,963 users who used both TT app and token. These include long-term Singapore residents and short-term visitors. Of all users, 5,569,572 were long-term Singapore residents, and 382,613 were short-term visitors. For reference, Singapore’s population for 2021 was 5,453,600 although this excludes citizens and permanent residents living abroad continuously for 12 months or more [9].

### Number of cases and number of registered TT devices

From 24 August 2021 to 17 February 2022, there were 466,849 new COVID-19 reported cases. This includes 449,362 (96.3%) local cases, with the remainder being imported cases. Of all cases, 435,451 cases (93.3%) had a registered TT device (either the app and/or token). Positive cases who agreed to upload their TT data for contact tracing had their data analysed for conversion rate and lead time savings. During the study period, 227,358 (52.2%) of positive cases with a registered TT device uploaded their data for contact tracing.

### Total number of QOs and HRWs issued and conversion rate

There were 346,588 HRWs automatically issued through TT, as well as 1,004,303 HRWs and 123,887 QOs issued through manual contact tracing supplemented by TT. Of these, 12,483 (3.6%) and 62,516 (6.2%), and 15,424 (12.5%) respectively subsequently tested positive for COVID-19 during the monitoring period. The average number of HRWs from TT per COVID-19 case was 5.35, and the average number of QOs from manual contact tracing per COVID-19 case was 6.54.

### Lead time savings from digital contact tracing

The duration from a confirmed COVID-19 case being registered on the central IT platform to automated issuance of HRW through TT was 18.3 h. In comparison, issuance of QO or HRWs through manual contact tracing supplemented by TT data was 28.1 h. This was a 9.8 h (34.9%) reduction in lead time.

## Discussion

Since the start of the pandemic, Singapore has managed to reduce COVID-19’s public health impact through a whole-of-government, whole-of-society approach. Central to this was quarantine and subsequently self-isolation of contacts traced by the government to reduce spread, morbidity and burden on healthcare systems. At the start of the Delta variant wave in August 2021, Singapore made a strategic shift from containment to mitigation of spread because it was deemed safer with a high national vaccination rate (As of 15 August 2021, 76% of the total population had received two vaccine doses, and this was more than 80% among those eligible) [10]. There was a short period of containment for the Omicron variant in December 2022 when it emerged, but this quickly reverted to a reduction of spread strategy once it was clear that the variant was not more severe. This posture continued through the Omicron variant wave that followed in January 2022, until the cessation of legal requirements for close contacts to self-isolate and self-test, in a shift towards personal responsibility.

We have described the utilisation, outputs and outcomes of the digital contact tracing system in Singapore, both for the automated identification of contacts and issuance of public health actions, but also as an adjunct to manual contact tracing efforts.

### High uptake rate of TT, but moderate upload compliance

The proportion of Singapore residents who had registered a TT-enabled device to their name was very high – close to 100%. Previous studies, both locally and overseas, have described public perception and challenges to the use of digital contact tracing tools in a pandemic [11–13]. The high uptake in Singapore was likely because the TT platform was linked to mandatory requirements to enter public spaces, in particular to check one’s vaccination status (i.e. through SE and vaccine-differentiated public health measures check-in). This nudge allowed for higher penetration in the population and improved its robustness, given the need for sufficient critical mass / proportion for such digital contact tracing tools to be effective, as it requires both the case and the contact to have an active device.

While this study did not cover the earlier period of the pandemic where manual contact tracing was done without the aid of digital tools, its subsequent introduction in March 2020 had already sharply reduced the time to process case and related contacts from 4 days to 1.5 days [personal communication]. This was critical in shoring up system efficienceis during the disease containment period when vaccines were still unavailable. Other studies also noted that digital contact tracing typically averted more cases during the super-critical phase of an epidemic when case counts were rising [14].

Over the period of our study, just above half of the cases agreed to upload their TT data. This was possibly due to the voluntary nature of uploads and because public health officers calling and verbally encouraging cases to do so became limited to those whom manual contact tracing was still conducted. In part, it could also have been due to pandemic fatigue especially once the actual and perceived risk of severe outcomes fell with vaccinations [15]. In the United States and Japan, low adoption rates were similarly seen in terms of upload of digital contact tracing data [16, 17]. Further studies are needed to better understand the reasons behind why some Singapore residents were not using TT, or not uploading their data after becoming positive cases. Addressing specific concerns or underlying factors may improve digital contact tracing data available and improve future efforts to reduce disease spread.

Singapore’s experience emphasises that engendering community trust for wide scale use is possible and necessary. Multiple assurances were provided regarding the integrity and security of data. It was strictly to be used for contact tracing and a narrow list of severe crimes [18]. A confirmed case would also need to provide consent for data to be uploaded, otherwise it would not be available to health authorities. After 25 days, the data was also automatically purged from all systems if unused. To further assuage concerns about the privacy and security of their contact tracing data, the open source protocol was published for public inspection. Moving forward, the role of ethical use frameworks and privacy safeguards should be further explored [19, 20].

### Limitations of TT

However, there are also technical limitations to digital contact tracing systems. For a close contact to be defined by TT, a high bar was set at 60 min of continuous close proximity to a case, as detected via Bluetooth. This duration and level of proximity was significantly higher than that required for spread to occur, especially given the higher transmissibility of new variants. This was done because of practical issues such as Bluetooth pinging across physical walls and barriers, leading to instances of inaccurate identification of contacts especially during periods of static activity, such as in offices or homes. Likewise, HRWs were used instead of QOs as they posed a lower level of imposition on the recipient in the event of such occurrences. Furthermore, individuals are also able to manually turn off Bluetooth functionalities on their phones, and this may lead to a reduction in true utilisation of digital contact tracing vis-à-vis the number of registered users. However, it was likely that most users kept it on, given the regular use of the TT app and token for vaccine differentiated entry into many public spaces.

### The role of digital contact tracing vis-à-vis manual contact tracing

Digital contact tracing has been adopted by various countries in this pandemic [3, 21]. Although Singapore is not unique in adopting new technologies to cope with the surge in cases, there are few other known instances of public health actions being automatically issued [22, 23]. More often, others have used such digital solutions to augment manual contact tracing [24].

The unique city-state context of Singapore and high adoption rate of TT, allows for early insights into how digital contact tracing solutions may allow partial or full replacement of traditional contact tracing approaches. This possibility has also been reflected in other studies [25, 26]. However, we show that manual contact tracing still provides a higher rate of conversion from contacts to cases, even for HRWs. This may suggest better precision than digital alternatives, although other factors were not captured, including differences in contagiousness, and the intensity of contact with source patients.

Given that manual contact tracing is labour intensive and slower, digital contact tracing remains a useful modality to augment situations where public health officers find themselves having limited resources [27]. Depending on digital contact tracing tools may free resources for other priorities [28, 29]. For instance, TT continued in the background for contact tracing of cases with the Delta variant, in parallel with manual contact tracing efforts for cases with the Omicron variant during the short period of containment. Adopting multiple tools for contact tracing has been shown to balance privacy concerns, efficiency concerns, and communication; and two ecological studies have showed incremental effectiveness of adding digital contact tracing to manual contact tracing [30, 31].

### Broader impact of digital contact tracing on Singapore’s COVID-19 pandemic control

Contact tracing played a key role in reducing COVID-19 spread, thereby preventing health systems in Singapore from being overwhelmed. Through time savings, digital contact tracing also improved efficiency of the contact tracing process, focusing manual efforts on higher priority cases, such as when the first cases of the Omicron variant emerged and it was still being characterised. Furthermore, as the platform was linked to vaccine-differentiated entry to public venues, there was wider adoption of digital contact tracing and indirectly encouraged vaccination uptake. All of these helped create a system to reduce the overall spread and severity of COVID-19 in Singapore.

## Conclusion

There has been increasing attention on how technology can be better used to augment public health efforts [32]. The promising outcomes in Singapore’s experience show that digital tools for contact tracing remain relevant and will likely play a key role in the management of future pandemics. The confluence of technology, urbanisation and globalisation warrants the development of novel approaches for more rapid, effective outbreak response and a transformation of traditional approaches to contact tracing.

## Data Availability

The datasets analysed are not publicly available. They have been deleted in keeping with obligations under the Singapore COVID-19 (Temporary Measures) Act. All protocols were approved by the Ministry of Health Singapore. When using the digital contact tracing application, participants were informed and consented for their data to be collected and analyzed by the Government of Singapore under the COVID-19 (Temporary Measures) Act. This study was conducted under the Infectious Diseases Act.

## References

[1] Louw C (2023). Digital public health solutions in response to the COVID-19 pandemic: comparative analysis of contact tracing solutions deployed in Japan and Germany. J Med Internet Res.

[2] Blasimme A, Ferretti A, Vayena E (2021). Digital contact tracing against COVID-19 in Europe: current features and ongoing developments. Front Digit Health.

[3] Whitelaw S, Mamas MA, Topol E, Van Spall HGC (2020). Applications of digital technology in COVID-19 pandemic planning and response. Lancet Digit Health.

[4] Lai SHS, Tang CQY, Kurup A, Thevendran G (2021). The experience of contact tracing in Singapore in the control of COVID-19: highlighting the use of digital technology. Int Orthop.

[5] Singapore, MOH. Ministry of Health (Singapore) COVID-19 statistics. 2022. https://www.moh.gov.sg/covid-19/statistics.

[6] Bay J, Kek J, Tan A, Hau CS, Yongquan L, Tan J, Quy TA. BlueTrace: a privacy-preserving protocol for community-driven contact tracing across borders. 2020. Retrieved from https://bluetrace.io/static/bluetrace_whitepaper-938063656596c104632def383eb33b3c.pdf.

[7] How does TraceTogether work? Government Technology Agency. Archived from the original on 31 May 2020. https://support.tracetogether.gov.sg/hc/en-sg/articles/360043543473-How-does-TraceTogether-work.

[8] Huang Z, Guo H, Lee YM, Ho EC, Ang H, Chow A (2020). Performance of digital contact tracing tools for COVID-19 response in Singapore: cross-sectional study. JMIR Mhealth Uhealth.

[9] Department of Statistics Singapore. Population and population structure. 2022. https://www.singstat.gov.sg/find-data/search-by-theme/population/population-and-population-structure/latest-data.

[10] Singapore, MOH. Update on Local COVID-19 situation and vaccination progress. 2022. August, 16, 2021 https://www.moh.gov.sg/news-highlights/details/update-on-local-covid-19-situation-and-vaccination-progress-(16-august-2021).

[11] Bannister-Tyrrell M, Chen M, Choi V, Miglietta A, Galea G (2023). Systematic scoping review of the implementation, adoption, use, and effectiveness of digital contact tracing interventions for COVID-19 in the Western Pacific Region. Lancet Reg Health West Pac.

[12] Tan LF, Tan MF (2021). Addressing endemic COVID-19 with high vaccination success: lessons from Singapore. J Am Med Dir Assoc.

[13] Lee JK, Lin L, Kang H (2021). The influence of normative perceptions on the uptake of the COVID-19 TraceTogether digital contact tracing system: cross-sectional study. JMIR Public Health Surveill.

[14] Burdinski A, Brockmann D, Maier BF (2022). Understanding the impact of digital contact tracing during the COVID-19 pandemic. PLOS Digit Health.

[15] Reicher S, Drury J (2021). Pandemic fatigue? How adherence to covid-19 regulations has been misrepresented and why it matters. BMJ.

[16] Ishimaru T, Ibayashi K, Nagata M, Tateishi S, Hino A, Tsuji M, Ando H, Muramatsu K, Fujino Y, CORoNaWork Project (2023). Factors associated with acceptance of a digital contact tracing application for COVID-19 in the Japanese working-age population. Nagoya J Med Sci.

[17] Cevasco KE, Roess AA (2023). Adaptation and utilization of a postmarket evaluation model for digital contact tracing mobile health tools in the United States: observational cross-sectional study. JMIR Public Health Surveill.

[18] Tang See Kit. Bill restricting use of TraceTogether data for serious crimes passed by Parliament. ChannelNewsAsia; 2021. https://www.channelnewsasia.com/singapore/bill-restrict-tracetogether-to-serious-crimes-passed-parliament-297611.

[19] Lo B, Sim I (2021). Ethical framework for assessing manual and digital contact tracing for COVID-19. Ann Intern Med.

[20] Saw YE, Tan EY, Liu JS, Liu JC (2021). Predicting public uptake of digital contact tracing during the COVID-19 pandemic: results from a nationwide survey in Singapore. J Med Internet Res.

[21] Sun HC, Liu XF, Du ZW, Xu XK, Wu Y (2021). Mitigating COVID-19 transmission in schools with digital contact tracing. IEEE Trans Comput Soc Syst.

[22] Jian SW, Cheng HY, Huang XT, Liu DP (2020). Contact tracing with digital assistance in Taiwan’s COVID-19 outbreak response. Int J Infect Dis.

[23] Daniore P, Ballouz T, Menges D, von Wyl V (2021). The SwissCovid Digital Proximity Tracing App after one year: were expectations fulfilled?. Swiss Med Wkly.

[24] Wacksman J (2021). Digitalization of contact tracing: balancing data privacy with public health benefit. Ethics Inf Technol.

[25] Cencetti G, Santin G, Longa A, Pigani E, Barrat A, Cattuto C, Lehmann S, Salathé M, Lepri B (2021). Digital proximity tracing on empirical contact networks for pandemic control. Nat Commun.

[26] Kozyreva A, Lorenz-Spreen P, Lewandowsky S, Garrett PM, Herzog SM, Pachur T, Hertwig R (2021). Psychological factors shaping public responses to COVID-19 digital contact tracing technologies in Germany. Sci Rep.

[27] Thomas Craig KJ, Rizvi R, Willis VC, Kassler WJ, Jackson GP (2021). Effectiveness of contact tracing for viral disease mitigation and suppression: evidence-based review. JMIR Public Health Surveill.

[28] Degeling C, Hall J, Johnson J, Abbas R, Bag S, Gilbert GL (2022). Should digital contact tracing technologies be used to control COVID-19? Perspectives from an Australian public deliberation. Health Care Anal.

[29] Albouy-Llaty M, Martin C, Benamouzig D, Bothorel E, Munier G, Simonin C, Guéant JL, Rusch E (2021). Positioning digital tracing applications in the management of the COVID-19 pandemic in France. J Med Internet Res.

[30] Chantziara S, Craddock IJ, Mccallum CH, Brigden ALC (2023). Views and needs of students, parents, and teachers on closed-circuit television, proximity trackers, and access cards to facilitate COVID-19 contact tracing in schools: thematic analysis of focus groups and interviews. JMIR Form Res.

[31] Pozo-Martin F, Beltran Sanchez MA, Müller SA, Diaconu V, Weil K, El Bcheraoui C (2023). Comparative effectiveness of contact tracing interventions in the context of the COVID-19 pandemic: a systematic review. Eur J Epidemiol.

[32] Soldano GJ, Fraire JA, Finochietto JM, Quiroga R (2021). COVID-19 mitigation by digital contact tracing and contact prevention (app-based social exposure warnings). Sci Rep.

